# Promoting the use of evidence (PROMOTE) in upper limb stroke rehabilitation: protocol for a multicentre, cluster-randomised, phase IV implementation trial

**DOI:** 10.1136/bmjopen-2026-119551

**Published:** 2026-08-19

**Authors:** Natasha A Lannin, Laura Jolliffe, Katharine Scrivener, Louise Ada, Catherine Dean, Tammy Hoffmann, Dominique A. Cadilhac, Elizabeth Lynch, Leonid Churilov

**Affiliations:** 1 School of Translational Medicine, Department of Neuroscience, Monash University, Melbourne, Victoria, Australia; 2 Bayside Health, Melbourne, Victoria, Australia; 3 School of Primary and Allied Health Care, Monash University, Melbourne, Victoria, Australia; 4 School of Medicine and Health, Macquarie University, Sydney, New South Wales, Australia; 5 Faculty of Medicine and Health, The University of Sydney, Sydney, New South Wales, Australia; 6 Institute for Evidence-Based Healthcare, Bond University, City of Gold Coast, Queensland, Australia; 7 Stroke and Ageing Research, School of Clinical Sciences at Monash Health, Monash University, Clayton, Victoria, Australia; 8 College of Health and Enablement, Flinders University, Bedford Park, South Australia, Australia; 9 Department of Medicine, The University of Melbourne, Melbourne, Victoria, Australia; 10 Melbourne Brain Centre, The Royal Melbourne Hospital, Melbourne, Victoria, Australia

**Keywords:** Stroke, REHABILITATION MEDICINE, Physical Therapy Modalities, Clinical trials, Implementation Science, Stroke Rehabilitation

## Abstract

**Introduction:**

Fewer than half of patients receive evidence-based upper limb rehabilitation after stroke. Implementation science interventions may improve use of guidelines by clinicians; however, it is not yet known whether they lead to evidence-based stroke rehabilitation practice. We aim to determine the effect and cost-effectiveness of a champion-led implementation package (PROMOTE) to increase adherence to best practice guidelines for upper limb rehabilitation after stroke.

**Methods and analysis:**

A multistate, multicentre, cluster-randomised, implementation phase IV trial with concealed allocation, blinded measurement and intention-to-treat analysis will be conducted. The PROMOTE package includes appointing an internal champion, local audit and feedback, interactive education tailored to gaps in knowledge and skill, and a decision-aid to support intervention selection. To be eligible, centres delivered rehabilitation to at least 20 stroke patients in the preceding year. Assuming 35% baseline adherence to guidelines, 14 centres (n=238) will be recruited to provide 80% power to detect a 30% absolute difference with a two-sided α=0.05. An embedded process evaluation and economic analysis will assess implementation and costs. The primary outcome is the proportion of people with stroke who are documented to receive guideline-recommended upper limb rehabilitation interventions by 6 months. Secondary outcomes include self-reported clinician behaviour change, upper limb therapy outcomes at the end of intervention, costs and cost effectiveness (eg, incremental cost per quality adjusted life year). Process evaluation outcomes include reach, adoption, implementation and maintenance (sustainability).

**Ethics and dissemination:**

Ethical approval was obtained from the Alfred Hospital Human Research Ethics Committee (approval number 89725 (NMA), 26 April 2023) with each participating site providing ethical clearance prior to the commencement of the trial. Findings will be reported in accordance with the Consolidated Standards of Reporting Trials extended for cluster-randomised trials and in peer-reviewed journals, and communicated to stakeholders and the public. Understanding and characterising the active ingredients of effective implementation will inform scalable strategies to improve stroke rehabilitation practice, health outcomes and the equitable delivery of evidence-based care.

**Trial registration number:**

The trial is currently recruiting and is registered at https://www.anzctr.org.au/.

ID: ANZCTR 12623000608662 (UTN U1111-1292-8705), 2 June 2023.

**STRENGTHS AND LIMITATIONS OF THIS STUDY**

- This is a fully powered, cluster-randomised phase IV implementation trial; randomising at the centre level, with matched pairing of centres and concealed, centrally-administered allocation, which protects against selection bias and minimises the risk of contamination between arms, although clinician movement between trial centres will be documented and accounted for in the analysis.
- While the design includes a cost-effectiveness analysis, embedded process evaluation and examination of post-implementation clinical outcomes, it does not assess sustainability of practice change after withdrawal of the champion and trial resources beyond the 6-month post-implementation phase.
- Centres vary in staffing, caseload, funding model and baseline guideline adherence; although matched-pair randomisation and pre-specified covariate adjustment will reduce confounding, residual centre-level heterogeneity may still influence implementation outcomes.
- The primary outcome relies on medical record audit and may be subject to documentation bias, although blinded assessors and standardised operational definitions will minimise this risk.
- All participating centres are within the Australian healthcare system and eligibility requires ≥20 stroke patients to be admitted per year; results may therefore not directly generalise to smaller, rural or resource-constrained services, to non-English speaking contexts or to healthcare systems with different funding models or staffing structures.

## Introduction

 There are over 440 000 Australians living with stroke,^1^ and approximately two-thirds experience upper limb impairments^2^ that affect their ability to perform everyday activities such as eating, dressing and washing.^3 4^ Upper limb impairments after stroke include not only muscle weakness but also reduced coordination and dexterity, compromising long-term independence.^5 6^ There is compelling evidence that delivering intensive, evidence-based upper limb rehabilitation will improve recovery,^7 8^ with guidelines recommending the amount and type of therapy (including constraint-induced movement therapy, repetitive task-specific motor training and electrical stimulation for strengthening).^9–17^

People with stroke who receive at least 2 hours a day^18^ of evidence-based upper limb rehabilitation^15 19^ are more likely to recover use of their arm. However, an Australia-wide audit of 103 rehabilitation services in 2024 showed that clinicians are not providing the recommended rehabilitation.^20^ Findings showed that services neither routinely provide the amount of upper limb rehabilitation recommended nor the most effective type of training, with little out-of-session practice. Importantly, the extent of this evidence-practice gap is not uniform: access to and receipt of rehabilitation by patients varies according to clinical factors such as stroke severity and upper limb impairment level, as well as socioeconomic and geographic factors, including rurality and healthcare system capacity.^21^ This multidimensional variation in rehabilitation access and delivery underscores the complexity of implementing guidelines at scale and highlights that effective implementation strategies must be responsive to the characteristics of clinicians, the healthcare recipients and the healthcare pathway. Similar challenges in translating guidelines into routine care have been observed in other areas of practice,^22 23^ reinforcing the importance of implementation interventions that are tailored to local context rather than applied uniformly.^24^

Existing implementation strategies in stroke rehabilitation, including audit-and-feedback approaches, have shown modest effects on clinician behaviour.^24^ However, audit and feedback alone is likely insufficient to drive sustained practice change when barriers to guideline uptake are multifactorial, encompassing gaps in the knowledge and skills of clinicians, limited access to resources, and cultural or organisational factors.^23 25^ What has been absent from the literature is a multicomponent, champion-led implementation package that combines education, skill-building, resourcing and feedback, tailored to the specific barriers identified at each centre, and tested in an adequately powered trial. Our Cochrane review confirmed this evidence gap,^24^ finding insufficient evidence to guide which strategies best support clinicians to deliver evidence-based neurorehabilitation.

Our pilot trial^26^ suggests that providing a champion to implement tailored support, including education and resources, improved guideline adoption. We additionally sought insights from clinicians and people with lived experience of stroke about the barriers and motivators for implementing upper limb rehabilitation guidelines,^27 28^ and tested methods of audit and feedback in rehabilitation.^29^ The pilot was a three-armed study testing: (1) PROMOTE+usual care versus (2) self-directed online resources+usual care versus (3) usual care alone. While we found that the champion-led package had a large effect, the self-directed package led to no greater increase in adherence than usual care. This suggests that passive dissemination, even when structured, was insufficient to drive practice change and that the active, champion-led components of PROMOTE were the likely drivers. Therefore, a larger adequately-powered trial is warranted to establish the effectiveness, cost-effectiveness and implementation characteristics of the full behaviour change package.

### Objectives

The primary objective is to evaluate the effectiveness of a champion-led implementation package versus usual standard rehabilitation on adherence to guideline recommendations for upper limb stroke rehabilitation in Australian hospitals.

Secondary objectives are:

- To evaluate the effectiveness of a champion-led implementation package versus usual standard rehabilitation on clinician implementation behaviour, upper limb ability, health benefits and harms.
- To evaluate the economic cost of implementing the package.
- To assess the fidelity of delivery of the package.
- To understand use of the package in terms of reach, adoption, implementation and maintenance.

## Methods

This protocol was developed and reported in accordance with the Standard Protocol Items: Recommendations for Interventional Trials (SPIRIT) 2025 statement. The PROMOTE intervention was co-developed with key stakeholders, including adults living with stroke, clinicians working in upper limb rehabilitation and partners from the Stroke Foundation. Practical guidance on intervention design and delivery was informed by qualitative work with stroke survivors and clinicians,^27 28^ and by ongoing input from a stakeholder advisory group chaired by Clive Kempson (stroke survivor) and clinician stakeholder team members to ensure feasibility and real-world relevance.

### Design

**Figure 1:**
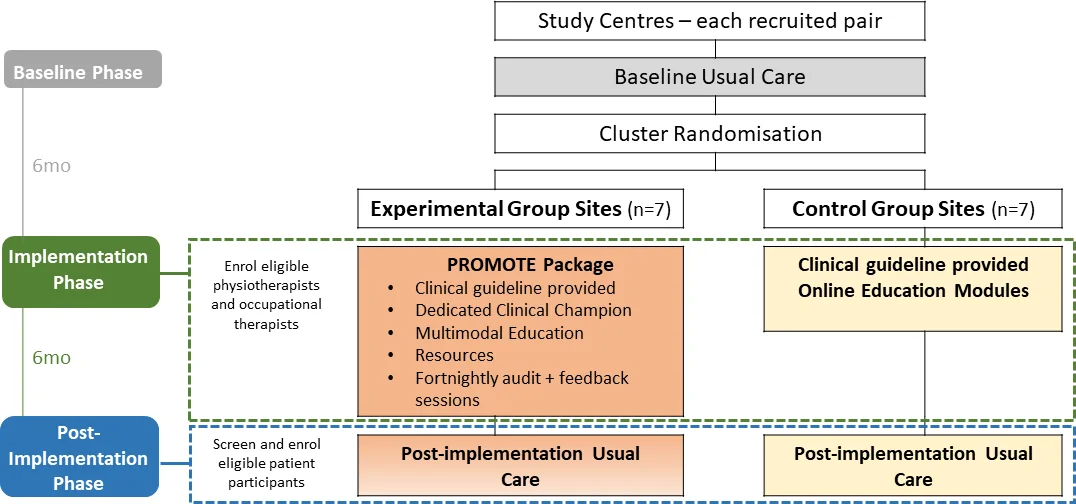
Trial flow diagram demonstrating the cluster-randomised design with a baseline usual care period. Participating centres are randomised in pairs according to readiness to commence. mo, months.

The PROMOTE trial is a two-arm, cluster-randomised, phase IV trial^30^ with concealed allocation, blinded measurement and intention-to-treat analysis (figure 1). The risk of contamination between intervention and control centres is minimised by the cluster-randomised design, whereby randomisation occurs at the centre level; however, in the event that staff move between an intervention and a control centre during the trial period, this will be documented and considered in the analysis. Randomisation will be undertaken centrally by an independent statistician to ensure allocation concealment, both clusters within each matched pair will be enrolled and complete baseline assessment before allocation to minimise selection bias, and the randomisation schedule will be concealed until immediately prior to the implementation phase to maintain temporal concealment. The trial includes three phases: a baseline usual care phase (12 months); a randomised implementation phase (6 months) during which implementation outcomes are assessed; and a post-implementation usual care phase (6 months) during which clinical outcomes are assessed. Only data from the baseline and implementation phases will be used for the primary analysis. Given the nature of the primary outcome, minimal amount of missing data are anticipated. Subject to the satisfiability of missingness at random assumption, depending on the amount of missing data, either complete case analysis or analysis with multiple imputations with chained equations will be undertaken followed by the sensitivity analysis investigating the plausibility of missingness at random assumption. The details will be reported in the statistical analysis plan finalised prior to the study data lock. During the post-implementation period, patients with stroke will be sequentially recruited at each centre and patient measures will be collected at hospital admission and after 4 weeks of rehabilitation (or at the end of rehabilitation if discharged sooner). Economic outcomes will be measured continuously over the randomised implementation phase. Service delivery costs will be obtained from the centres for each trial phase (ie, to determine the implementation delivery costs).

### Participants, interventions and outcomes

#### Setting

Inpatient rehabilitation centres that deliver upper limb rehabilitation will be eligible to participate if they (1) employ at least one occupational therapist and/or physiotherapist, (2) receive >20 stroke referrals annually, and (3) have a level of adherence to guidelines <50% per stroke survivor in the previous Stroke Foundation rehabilitation services audit.^20^ Centres will be described by location, hours of therapy per bed (intensity of rehabilitation), funding (private vs public), staffing and caseload volume, and Australasian Rehabilitation Outcomes Centre data (eg, Functional Independence Measure (FIM) efficiency).

#### Eligibility criteria

Clinician participants will be occupational therapists and/or physiotherapists who work on participating wards and prescribe and deliver upper limb rehabilitation. Inpatients with stroke undergoing upper limb rehabilitation will be eligible to participate during the post-implementation phase if they are over 18 years old, have sustained a stroke in the previous 6 months, are able to read and understand spoken English to a level where they can understand the participant information sheet, can complete the consent form with/without an interpreter or next of kin, and have stroke-related difficulty using their upper limb that warrants rehabilitation (ie, have a minimum of 5° active finger or wrist extension and a maximum ability of 30 blocks/min on the Box and Block test).

#### Interventions

Experimental centres will receive a champion-led implementation package (PROMOTE) tailored to address each centre’s identified barriers^31^ by an experienced implementation scientist (NAL). The PROMOTE package includes several components:

- Clinical practice guidelines: a copy of Clinical Guidelines for Stroke Management.^11^
- Champion: a dedicated, experienced clinician who advocates, encourages, coaches, mentors, educates, trains, persuades and motivates other clinicians.^32^ Champions will be appointed and selected by site management, drawing from existing experienced clinicians already working within the site. They will be supported during the implementation phase by a community of practice facilitated by an experienced clinician and implementation scientist (LJ).
- Education: a multimodal package designed to support knowledge gain, skill development and transfer of learning into clinical care.^33^ The package includes skills training (in-person), fortnightly micro-learning sessions (online, live), and online education modules (self-directed), posters and written manuals to train clinicians in clinical guideline-recommended interventions.^34–36^
- Resources: electrical stimulation machines, equipment kits to deliver the Graded Repetitive Arm Supplementary Program (GRASP),^37^ mirror boxes, constraint-induced movement therapy mitts, treatment tables and care pathways to simplify clinical reasoning.
- Fortnightly audits of two current stroke inpatients presented to stimulate team discussion on guideline use (ie, proportion of recommended guidelines met, number of sessions and team challenges).^29^

Control centres will receive a copy of the Clinical Guidelines for Stroke Management. To standardise access to clinical education during the implementation phase, control centres will also receive access to the online education modules for self-directed completion. Fidelity to the intervention components will be monitored through champion activity log sheets, clinician education attendance and module completion records. Full details of both intervention arms are described in accordance with the Template for Intervention Description and Replication checklist (online supplemental file C).

#### Outcomes

The primary outcome is the proportion of inpatients with stroke with upper limb impairment who are documented to receive the recommended amount and type of upper limb rehabilitation during their admission. Guideline-recommended care was defined per the Stroke Foundation’s Clinical Guidelines for Stroke Management^11^ as ≥2 hours/day of active upper limb practice^18^ delivered using at least one of the constraint-induced movement therapy, repetitive task-specific training or electrical stimulation for strengthening; operational definitions for each are provided in online supplemental file D.

Secondary outcomes include change in clinician implementation behaviour as measured on a Theoretical Domains Framework questionnaire^38^ collected during baseline and implementation phases. Secondary clinical outcomes will be collected during the post-implementation phase, including change in upper limb ability (end of treatment minus admission) measured using the Box and Block test^39^ and Action Research Arm test.^40^ Health benefits will be estimated using the self-rated quality of life tool, EuroQol 5-Dimensions (EQ-5D-5L)^41^ and a preference-based single index score, using Australian preference weights, will be derived.^42^

Activity-based costing will be used to quantify the economic cost of implementing the interventions as an average cost per patient, inclusive of training, supervision and overall rehabilitation costs for eligible patients. Patient-level costs will be measured in $A and reported from a single reference year based on a societal perspective (including direct and indirect costs). Incremental cost per quality-adjusted life year gained will be estimated from the EQ-5D-5L data (change baseline to 4 weeks) and the total costs of intervention delivery compared across groups. Economic modelling will be used where relevant.

A process evaluation will be undertaken to collect intervention data and understand the use of the PROMOTE package within experimental centres from the perspective of clinicians, managers and people with lived experience. Deductive coding of transcribed interviews will be analysed alongside intervention data and findings will be reported according to the RE-AIM framework (Reach, Effectiveness, Adoption, Implementation and Maintenance).^43^

Because this phase IV trial implements only guideline-recommended interventions already expected to be delivered as part of routine care, the risk of study-related harm is low. Harms were defined a priori as any adverse events or unintended consequences occurring during usual rehabilitation and anticipated to potentially include falls, soft tissue injuries or unexpected deterioration in neurological or medical status. Assessment of harms will be the responsibility of the treating clinicians and will be logged and reported in accordance with institutional and ethics requirements.

#### Sample size

With a baseline adherence to guidelines of 35%,^44^ assuming control centres will increase this adherence to 40% and experimental centres to 70%, that is, an absolute difference of 30%, we will recruit 14 centres (clusters) evenly split between experimental and control arms. The intraclass correlation coefficient (ICC) observed in our pilot was <0.01.^26^ Assuming more conservative settings of ICC=0.05, a coefficient of variation of cluster sizes of 0.99 and no correlation between baseline measures and the outcome, recruiting 7 clusters per arm with an average 17 patients per cluster (a total sample size of 238 patients, 119 per arm) would yield power of 0.8 to observe an absolute difference of at least 30% with a two-sided alpha=0.05.

#### Recruitment

Centres will be recruited across Victoria, New South Wales and South Australia and randomised in matched pairs to receive either the PROMOTE implementation package or online education to support guideline implementation.

### Assignment of interventions

#### Randomisation and blinding

Pairs of centres matched on throughput of stroke patients, level of adherence to guidelines reported to Stroke Foundation, and metropolitan or rural will be randomised to experimental or control (1:1) by an independent statistician. Centres will be notified by the principal investigator of their randomised group following the baseline usual care phase and within 4 weeks of an agreed date before commencing the implementation phase.

#### Blinding

All outcomes will be measured by researchers blinded to centre (cluster) allocation. Statistical analysis will be carried out by a statistician blinded to randomisation.

### Data collection and analysis

Data collection commenced in January 2024 and is ongoing (expected to be completed by August 2026, with results available from December 2026).

#### Data collection

Each participating site will appoint a site coordinator responsible for managing participant flow and overseeing local data collection. Blinded outcome assessors will access data either through secure login (primary outcome) or on-site visits to perform assessments (secondary outcomes). All study data will be stored in site-specific, encrypted, access-restricted Microsoft Teams folders, with de-identified data maintained separately from any re-identification keys, ensuring that only authorised personnel at each site can access their own secure database. At the completion of data collection, the de-identified dataset of each site will be securely transferred to Monash University (sponsor) and entered into a REDCap electronic data capture tool hosted at Monash University.^45 46^

#### Statistical analyses

Intention-to-treat analyses will be conducted. A statistical analysis plan will be finalised prior to locking the study database. For the primary analysis of the binary outcome of whether guideline recommendations were received by the participant or not, a three-level mixed effect logistic regression model with the intervention as independent variable and individual centre and the matched pair of centres used for randomisation as nested random effects will be used. The model will be adjusted for the following pre-specified covariates: baseline participant’s age and FIM score, as well as the cluster case volume and proportion of cases with guideline-recommended care provided during the baseline usual care stage. The intervention effect will be presented as adjusted odds ratio and standardised risk difference with respective 95% CI as per US Food and Drug Administration Guidance for Industry ‘Adjusting for Covariates in Randomized Clinical Trials for Drugs and Biological Products’. Relevant secondary outcomes will be analysed by appropriate mixed-effect regression models with the random effects specified as per the primary analysis adjusted for baseline participant’s age and baseline value of the respective outcome variable.

The economic evaluation will be conducted in accordance with the Consolidated Health Economic Evaluation Reporting Guidelines. The incremental cost-effectiveness ratio (ICER) will be calculated relative to the control group and will be reported as a cost per quality-adjusted life year gained. An ICER below $A 50 000 will be considered cost-effective. Acceptability curves of the probability that an intervention is cost-effective as a function of willingness to pay will also be plotted. Appropriate one-way sensitivity and multivariable uncertainty analyses or modelling will be performed to assess the robustness of results.

### Monitoring

All adverse events will be logged during the trial and rated by site principal investigators for likelihood of trial relatedness prior to being forwarded to the data monitoring clinician for review.

#### Data monitoring clinician

Data quality and safety monitoring will be overseen by an independent, experienced physiotherapist and clinical trialist (David Berlowitz, Melbourne University). He will be responsible for reviewing data and endpoints on a regular basis and monitoring and actioning any serious trial-related adverse events.

### Patient and public involvement

When developing the protocol, the chief investigator met with the Monash University Brain Recovery and Rehabilitation Research Consumer Advisory Group, a collaborative group of researchers and consumers dedicated to embedding meaningful lived experience of stroke within clinical research. During a presentation to this group, the team discussed the design of the PROMOTE trial, including its cluster-randomised structure and the wording of inpatient participant-facing materials. Following protocol development, co-investigator Clive Kempson (person with lived experience of stroke) was appointed to chair a PROMOTE Trial Patient and Public Involvement (PPI) committee, and provides representation on the Trial Management Committee. This PPI committee meets regularly during trial conduct and reviews all participant-facing documents and dissemination materials.

## Ethics

Ethical approval was granted through the National Mutual Acceptance Scheme by Alfred Hospital Human Research Ethics Committee (approval number 89725(NMA), 26 April 2023). Each participating centre has obtained ethical approvals from their Institutional Research Ethics Committee (online supplemental file A). The trial was registered at ANZCTR 12623000608662 (2 June 2023). Written informed consent will be obtained from clinician participants prior to randomisation (online supplemental file B), and from patient participants prior to baseline assessment. This study will be conducted in compliance with the principles outlined in the World Medical Association’s Declaration of Helsinki.^47^

## Supplementary material

10.1136/bmjopen-2026-119551 online supplemental file 1

10.1136/bmjopen-2026-119551 online supplemental file 2

10.1136/bmjopen-2026-119551 online supplemental file 3

10.1136/bmjopen-2026-119551 online supplemental file 4
