# Supplementary material for: Promoting the use of evidence (PROMOTE) in upper limb stroke rehabilitation: protocol for a multicentre, cluster-randomised, phase IV implementation trial
Source: BMJ Open. 2026 Aug 19;16(8):e119551. doi: 10.1136/bmjopen-2026-119551 (PMC13504908; doi:10.1136/bmjopen-2026-119551)
Supplement: Supplementary file 1 [file bmjopen-16-8-s001.pdf]

Supplementary File C

Table C1: Template for Intervention Description and Replication (TIDieR) checklist outlining the PROMOTE Trial Intervention components.

Promoting the use of evidence (PROMOTE) in upper limb stroke rehabilitation: multicentre, cluster-randomised, implementation Phase IV trial

Item	TIDieR Item	Experimental: PROMOTE Package	Control: Usual Care + Online Education
1	**Brief name**	PROMOTE Package: a champion-led implementation package to increase adherence to evidence-based upper limb rehabilitation guidelines after stroke.	Usual standard rehabilitation with online education access.
2	**Why**	Grounded in implementation science and behaviour change theory (COM-B and Theoretical Domains Framework). The package targets identified barriers to guideline uptake through:<br>• A dedicated internal champion to advocate, educate, coach, and motivate clinicians<br>• Tailored education to close knowledge and skill gaps<br>• Audit-and-feedback to stimulate reflection and team discussion<br>• Resources to enable delivery of recommended interventions, including provision of all necessary equipment to deliver guideline recommended interventions<br>Informed by qualitative work with stroke survivors and clinicians, and co-developed with a stakeholder advisory group.	• Provision of clinical practice guidelines<br>• Online self-directed education to maintain ethical standards of care and standardise educational access across groups.<br>• No active implementation support is provided.
3a	**What (Materials)**	For clinicians:<br>• Copy of the Clinical Guidelines for Stroke Management (Stroke Foundation, 2023)<br>• Access to online education modules (self-directed)<br>• In-person skills training (CIMT, electrical stimulation) materials and written manuals<br>• Point-of-care demonstration videos<br>• Fortnightly micro-learning sessions (online, live)<br>• Posters and care pathways to simplify clinical reasoning<br>• Staff survey to identify barriers and enablers to guideline use (administered at baseline to tailor program)<br>• Champion log-sheets to record time and activities during the implementation phase<br>Equipment and resources:<br>• Electrical stimulation machines<br>• GRASP equipment kits<br>• Mirror boxes<br>• Constraint-induced movement therapy (CIMT) mitts and practice boxes with matched exercises<br>• Treatment tables<br>For audit and fidelity monitoring:<br>• Fortnightly audit of therapy programs received by two current inpatients, and feedback sessions facilitated by the Champion.	• Copy of the Clinical Guidelines for Stroke Management (Stroke Foundation, 2023)<br>• Access to online education modules (self-directed; same modules provided to the Experimental group)

**3b**	**What (Procedures)**	The PROMOTE package is tailored to each centre's identified barriers by an experienced implementation scientist (NL) and delivered during a 6-month implementation phase. Key procedures include:<br>• Appointment of an internal Champion at each experimental centre (an experienced clinician already employed at the centre selected by site management against a standard position description), provided with additional training and support<br>• Champion supported throughout with weekly meetings with research team (NL/LJ/KS) and via a community of practice with the research team (LJ) and other champions<br>• Multimodal education delivered via: in-person skills training (workshops and point-of-care demonstrations provided by research team (NL)); live fortnightly micro-learning (online) provided by research team (KS); self-directed online modules<br>• Fortnightly audit of two current or recently discharged stroke patients per center, with structured team feedback addressing: proportion of patients receiving recommended guidelines, number of sessions, stroke severity, and team challenges<br>• Resources provisioned to enable delivery of recommended interventions (e.g., electrical stimulation, GRASP, CIMT)	Centres receive usual rehabilitation practice. No champion is appointed and no active implementation support, education sessions, or audit-and-feedback activities are provided beyond the materials listed above.
**4**	**Who provided**	Implementation scientist: conducts tailored barrier analysis and customises the PROMOTE package for each center prior to and during the implementation phase (NL), supports fortnightly audit-and-feedback activities (NL and LJ) and facilitates a community of practice for the clinical champions to meet throughout the implementation phase (LJ).<br>• Clinical champion: a clinician already employed at each experimental center (occupational therapist or physiotherapist), selected for their experience and credibility with peers; provided with additional structured training (2 days) and ongoing mentoring and support from the research team (NL, LK, & KS) and champion community of practice throughout the 6-month implementation phase.<br>• Experienced educator: supports education delivery and resource development and provision (NL/KS/LA/CD)	No specific intervention provider beyond usual care treating rehabilitation clinicians (occupational therapists and physiotherapists) delivering usual care.
**5**	**How**	• In-person: skills training workshops; champion coaching and mentoring<br>• Online (live): weekly micro-learning sessions (group format)<br>• Online (self-directed): education modules (individual)<br>• Team-based: fortnightly audit-and-feedback presented to the clinical team<br>• Community of practice: champion support network (online/remote)	• Online (self-directed): education modules (individual)
**6**	**Where**	Inpatient rehabilitation centres in Victoria, New South Wales, and South Australia, Australia. Centres must employ ≥1 occupational therapist and/or physiotherapist and receive >20 stroke referrals annually. Both metropolitan and rural centres are included.	
**7**	**When and how much**	Six-month implementation phase (≥180 days):<br>• In-person skills training: delivered within first 30 days of commencement of the implementation phase (workshop format)<br>• In-person mentorship, modelling and clinical reasoning support to deliver guideline-recommended upper limb therapy provided by Champion (available 0.5FTE)	Six-month implementation phase (≥180 days):<br>• Online education modules: self-paced, completed during the implementation phase

		• Fortnightly micro-learning sessions: approximately 26 sessions over 6 months (online, live, 15 minutes per session; recorded to allow staff to review independently)<br>• Online education modules: self-paced, completed during the implementation phase<br>• Fortnightly audit-and-feedback: approximately 6 cycles over 6 months, each covering two stroke inpatients; feedback presented to the clinical team by Champion<br>• Champion support and community of practice: ongoing throughout 6 months	
**8**	**Tailoring**	The PROMOTE package is explicitly tailored to each center's identified barriers to guideline uptake, informed by the COM-B framework and Theoretical Domains Framework. Tailoring is conducted by the implementation scientist (NL) prior to and during the implementation phase, based on local audit data, stakeholder input, and knowledge/skill gap assessment. Specific components (e.g., focus of education, type of resources provided) are adapted to address center-specific needs.	No tailoring. Standard materials provided uniformly to all control centers.
**9**	**Modifications**	Any modifications to the intervention during the trial will be documented and reported in the final publication.	
**10a**	**How well (Planned)**	Intervention adherence will be assessed through multiple measures, including:<br>• Reach: proportion of eligible clinicians and patients engaged,<br>• Adoption: uptake of the PROMOTE package components at each center including records of clinician attendance at education sessions and online records of module completion; Champion log-sheets recording time spent and activities completed during the implementation phase.<br>• Implementation: Medical record audits at 0, 6, and 12 months by blinded assessors, to assess clinician adherence to guidelines plus video recording of one therapy session per patient per week (maximum four per patient), independently audited to evaluate dose (repetitions, time on task, total time) and content against National Clinical Practice Guidelines<br>• Maintenance: sustainability of practice change post-implementation<br>A process evaluation embedded within the trial will assess broader implementation using the RE-AIM framework (Reach, Adoption, Implementation, Maintenance), drawing on clinician surveys, qualitative interviews with clinicians and inpatients, and intervention activity data.	Intervention adherence:<br>• Online records of module completion<br>Primary outcome data (medical record audit) collected by blinded assessors.
**10b**	**How well (Actual)**	To be reported following trial completion.	

Abbreviations: CIMT = constraint-induced movement therapy; COM-B = Capability, Opportunity, Motivation–Behaviour model; GRASP = Graded Repetitive Arm Supplementary Program; RE-AIM = Reach, Effectiveness, Adoption, Implementation, Maintenance; TDF = Theoretical Domains Framework.
