# Supplementary material for: Promoting the use of evidence (PROMOTE) in upper limb stroke rehabilitation: protocol for a multicentre, cluster-randomised, phase IV implementation trial
Source: BMJ Open. 2026 Aug 19;16(8):e119551. doi: 10.1136/bmjopen-2026-119551 (PMC13504908; doi:10.1136/bmjopen-2026-119551)
Supplement: Supplementary file 2 [file bmjopen-16-8-s002.pdf]

Supplementary File D

Table D1: Medical Record File Audit: Arm Function items.

Promoting the use of evidence (PROMOTE) in upper limb stroke rehabilitation: multicentre, cluster-randomised, implementation Phase IV trial.

Questions consistent with Stroke Foundation (Australia) Stroke Services Spot Audit Arm Function Module. Available from: https://informme.org.au/stroke-data/spot-audits

AuSDaT ref	Question	Response Options
**PRIOR TO STROKE**		
6.470	Functional status prior to stroke?	
6.540	Living arrangements at admission	Home (alone); Home (with others); Supported accommodation e.g. nursing home, hostel; Other
**ON ADMISSION TO REHABILITATION BED**		
-	Functional Independence Measure (FIM™) at Rehabilitation Admission	Item scores, taken from Australian Rehabilitation Outcomes Centre (AROC) database for case
-	Admission ICD10 code	
7.550	Type of stroke	TIA; Ischaemic; Haemorrhage; Undetermined
L9.000	On admission were any of the following impairments present:	
9.000	Sensory deficit	Yes / No / Not documented
9.010	Cognitive deficit	Yes / No / Not documented
9.020	Visual deficit	Yes / No / Not documented
9.030	Perceptual deficit	Yes / No / Not documented
-	Speech/communication impairment	Yes / No / Not documented
9.061	Arm deficit	Yes / No / Not documented
**ARM ASSESSMENT**		
9.460	Was the patient's upper limb (UL) assessed?	Yes / No / Not documented
L9.47	List UL Assessment measures used:	Free text
9.470	Upper limb component of the Motor Assessment Scale: UL-MAS	Yes / No
9.480	9 hole peg - test: 9HPT	Yes / No
9.490	Any other UL assessments	Yes / No
**ARM REHABILITATION / MANAGEMENT**		
10.500	Was the case seen by an Occupational Therapist (OT)?	Yes / No
10.510	OT Date commenced	DDMMYYYY; unknown
10.530	OT Time of first contact	hh:mm; unknown
-	OT Date completed	DDMMYYYY; unknown
10.450	Was the case seen by a Physiotherapist (PT)?	Yes / No
10.460	PT Date commenced	DDMMYYYY; unknown
10.48	PT Time of first contact	hh:mm; unknown
-	PT Date completed	DDMMYYYY; unknown
9.510	Did the case receive constraint-induced movement therapy (CIMT)	Yes / No
-	CIMT Date commenced	DDMMYYYY; unknown
-	CIMT Therapist	OT / PT / EP / AHA / Other
-	CIMT Date completed	DDMMYYYY; unknown
-	Number of CIMT sessions completed	
9.520	Repetitive task-specific training (TST)	Yes / No
-	Date repetitive TST commenced	DDMMYYYY; unknown
-	Repetitive TST Therapist	OT / PT / EP / AHA / Other
-	Date repetitive TST completed	DDMMYYYY; unknown
-	Number of repetitive TST sessions completed	
9.530	Mechanically assisted training (robotics)	Yes / No
-	Robotics Date commenced	DDMMYYYY; unknown
-	Robotics Therapist	OT / PT / EP / AHA / Other
-	Robotics Date completed	DDMMYYYY; unknown
-	Number of Robotics sessions completed	

Supplementary File D

Table D1: Medical Record File Audit: Arm Function items.

AuSDaT ref	Question	Response Options
**ARM REHABILITATION / MANAGEMENT**		
-	Electrical Simulation of UL (ES)	Yes / No
-	ES Date commenced	DDMMYYYY; unknown
-	ES Therapist	OT / PT / EP / AHA / Other
-	ES Date completed	DDMMYYYY; unknown
-	Number of ES sessions completed	
9.540	Other UL therapy	List, Yes / No
-	Other therapy, Date commenced	DDMMYYYY; unknown
-	Other therapy, Therapist	OT / PT / EP / AHA / Other
-	Other therapy, Date completed	DDMMYYYY; unknown
-	Other therapy, number of sessions completed	
**COMPLICATIONS**		
L11.01	***On admission*** were any of the following complications present:	
11.070	Shoulder subluxation	Yes / No
11.080	Shoulder pain	Yes / No
11.100	Contracture	Yes / No
L11.16	Did the case experience any of the following complications **during** their admission:	
11.230	Shoulder pain	Yes / No
11.240	Shoulder subluxation	Yes / No
11.350	Contracture	Yes / No
-	Any other Complications (LIST):	
11.370	Were any of the above complications severe (i.e. incapacitating, life threatening and/or prolong hospital admission and patient acuity)?	Yes / No / Unknown
**ON DISCHARGE FROM REHABILITATION BED**		
14.080	Date of discharge	DDMMYYYY
14.160	What is the discharge destination/mode	Discharge/transfer to (an)other acute hospital<br>Discharge/transfer to a residential aged care service, unless this is the usual place of residence<br>Statistical discharge - type change<br>Left against medical advice/discharge at own risk<br>Died<br>Other<br>Usual residence (e.g. home) with support<br>Usual residence (e.g. home) without support<br>Inpatient rehabilitation<br>Transitional care service
14.161	Please specify (if residential aged care)	Low level residential care;<br>High level residential care
**Dependency on Discharge**		
14.250	Functional status at discharge?<br>(mRS) Score 0-6	0-6 (unknown or derived if needed)
-	Functional Independence Measure (FIM™) at Rehabilitation Discharge	Item scores, taken from Australian Rehabilitation Outcomes Centre (AROC) database for case
