# Supplementary material for: Promoting the use of evidence (PROMOTE) in upper limb stroke rehabilitation: protocol for a multicentre, cluster-randomised, phase IV implementation trial
Source: BMJ Open. 2026 Aug 19;16(8):e119551. doi: 10.1136/bmjopen-2026-119551 (PMC13504908; doi:10.1136/bmjopen-2026-119551)
Supplement: Supplementary file 3 [file bmjopen-16-8-s003.pdf]

Supplementary File A

List of Participating Sites and Approving Ethics Committees

Promoting the use of evidence (PROMOTE) in upper limb stroke rehabilitation: multicentre, cluster-randomised, implementation Phase IV trial

Lead Committee	Alfred Hospital Ethics Committee	NMA: 89725<br>Approved 29-Nov-2022
Alfred Health: Caulfield Hospital	Alfred Hospital Ethics Committee	569/22<br>Approved 26-Apr-2023
Austin Health: Royal Talbot Hospital; Heidelberg Repatriation Hospital	Austin Health Human Research Ethics Committee	SSA/89725/Austin-2023<br>Approved 10-Oct-2023
Balmain Hospital	Sydney Local Health District Ethics Review Committee	X24-0082<br>Approved 20-May-2024
Bendigo Health	Bendigo Health Human Research Ethics Committee	BH-2023-376715<br>Approved 01-Aug-2023
Eastern Health: Peter James Centre	Eastern Health Human Research Ethics Committee	S23-067-89725<br>Approved 10-Oct-2023
Flinders Medical Centre	Southern Adelaide Clinical Human Research Ethics Committee	2023/SSA00575<br>Approved 13-Dec-2023
Hornsby Ku-Ring-Gai Hospital	Northern Sydney Local Health District Human Research Committee	2024/STE00012<br>Approved 10-May-2024
Peninsula Health: Golf Links Road; The Mornington Centre,	Peninsula Health Human Research Ethics Committee	SSA/89725/PH-2023<br>Approved 07-SEP-2023
Queen Elizabeth Hospital	Central Adelaide Local Health Network Human Research Ethics Committee	MYIP19093<br>Approved 15-Aug-2024
Rankin Park Hospital	Hunter New England Local Health District Human Research Ethics Committee	2023/STE01918<br>Approved 12-Oct-2023
Ryde Hospital	Northern Sydney Local Health District Human Research Ethics Committee	2023/STE04634<br>23-Apr-2024
Wagga Wagga Base Hospital	Murrumbidgee Local Health District	2023/STE01917<br>Approved 11-Sept-2023
Wallaroo Rehabilitation Service	Department for Health and Wellbeing Human Research Ethics Committee	2023/SSA00674<br>Approved 20-Mar-2024
