# Supplementary material for: Promoting the use of evidence (PROMOTE) in upper limb stroke rehabilitation: protocol for a multicentre, cluster-randomised, phase IV implementation trial
Source: BMJ Open. 2026 Aug 19;16(8):e119551. doi: 10.1136/bmjopen-2026-119551 (PMC13504908; doi:10.1136/bmjopen-2026-119551)
Supplement: Supplementary file 4 [file bmjopen-16-8-s004.pdf]

Supplementary File B

Copy of Participant Information Sheet.

Promoting the use of evidence (PROMOTE) in upper limb stroke rehabilitation: multicentre, cluster-randomised, implementation Phase IV trial

Interventional Study - *Adult providing own consent*

[Insert site name]

**Title**	PROMOTE: A Cluster-Randomised
**Short Title**	PROMOTE
**Project Sponsor**	Monash University
**Coordinating Principal Investigator**	Prof Natasha Lannin
**Site Principal Investigator**	*[Site PI Name and Affiliation Here]*
**Site Location**	*[Location]*

Part 1 What does my participation involve?

1 Introduction

You are invited to take part in this research project. This is because you are an occupational therapist, physiotherapist, exercise physiologist or allied health assistant working in a centre that delivers upper limb rehabilitation. The PROMOTE Trial is a cluster, randomised controlled trial which means that centres (hospitals) are randomly allocated to a group: therapists at these centres will be provided with one of the two education packages.

This Participant Information Consent Form tells you about the research project. It explains the implementation interventions that are involved. Knowing what is involved will help you decide if you want to take part in the research.

Please read this information carefully. Ask questions about anything that you don't understand or want to know more about.

Participation in this research is voluntary. If you don't wish to take part, you don't have to.

If you decide you want to take part in the research project, you will be asked to sign the consent section. By signing it you are telling us that you:

- Understand what you have read
- Consent to take part in the research project
- Consent to take part in the implementation interventions that are described
- Consent to the use of your medical notes as described.

You will be given a copy of this Participant Information and Consent Form to keep.

2 What is the purpose of this research?

The aim of this study is to evaluate the clinical benefit and cost-effectiveness of a champion-led implementation package (PROMOTE) to increase clinician adherence to known best practice guidelines for arm and stroke rehabilitation. While the main purpose is to understand this from the perspective of clinicians, we also want to understand whether it will change what therapy is provided to patients and the improvements patients may experience. A process evaluation will also be conducted alongside the main trial to understand the challenges faced during arm and hand therapy.

Project outcomes will provide important recommendations on how to support rehabilitation clinicians nationally for the benefit of stroke survivors.

Supplementary File B

Copy of Participant Information Sheet.

Promoting the use of evidence (PROMOTE) in upper limb stroke rehabilitation: multicentre, cluster-randomised, implementation Phase IV trial

3 What does participation in this research involve?

By agreeing to take part in this study, you are consenting to a research team member accessing the medical notes you have written for the patients seen for upper limb rehabilitation. Your medical entries will be retrospectively audited against set criteria at three time points (prior to the intervention phase, after the intervention phase, and 6-months after the intervention phase has finished). This will require no time or additional tasks to you. The researcher involved in reading your notes will be a staff member or honorary staff member at your organisation, and a waiver of patient consent has been approved by the ethics committee for these audits to occur.

This study involves two different groups. Each group (or site cluster) will be provided with a package of knowledge translation strategies during the intervention phase. If you agree to take part in this study, your work site (as a cluster) will be assigned to the support you will receive.

> **Group One:** If your centre is assigned to this group, you will be invited to participate in fortnightly feedback meetings and fortnightly education sessions (10 minutes each) for the duration of the intervention period (6 months). You will also be invited to participate in in-services, coaching and mentoring sessions, online education, access to pre-planned treatment plans, discussion of evidence, and point of care videos to support your practice.
>
> **Group Two:** If your centre is assigned to this group, you will have education material made available to you, this may include: education handouts for patients, evidence summary material, online educational modules to support your clinical practice.

At two times during the study, we will ask you to complete a short 10-minute survey (online) about your perceptions of the education and of upper limb rehabilitation and the Stroke Guidelines. We are also interested to know your thoughts on the knowledge translation package of interventions that you were provided with and therefore you will also be invited to participate in qualitative discussions (interview or focus group) about your perceptions.

By agreeing to take part in this study, you and a research team member will screen your current patient caseload. Patients who have a diagnosis of stroke and have upper limb rehabilitation goals will be eligible. For each patient that consents to participating in the study during the post-intervention phase, you agree to complete a therapy log book during the study to write down the duration of each upper limb session you have with that patient over the 4-week period. It is anticipated that this will require a total of 10 minutes of your time at each week (less than 1 hour in total). If a patient that has consented the study is to be discharged before the end of the study period (4 weeks per patient), you agree to informing a research team member (via email, phone or in person) as soon as you are made aware. It is anticipated that this will take approximately 5-10 minutes of your time.

There are no additional costs associated with participating in this research project, nor will you be paid. All resources required as part of the research project will be provided to you free of charge.

4 Other relevant information about the research project

Overall, a total 14 centres that deliver upper limb rehabilitation in the health services of New South Wales (NSW), Victoria and South Australia (SA) will take part in this study.

This project involves researchers from a number of organisations working in collaboration; the project is led by Professor Natasha Lannin who is based at Monash University and Alfred Health (Melbourne). This research has been funded by the Medical Research Future Fund (MRFF).

Supplementary File B

Copy of Participant Information Sheet.

Promoting the use of evidence (PROMOTE) in upper limb stroke rehabilitation: multicentre, cluster-randomised, implementation Phase IV trial

5 Do I have to take part in this research project?

Participation in any research project is voluntary. If you do not wish to take part, you do not have to. If you decide to take part and later change your mind, you are free to withdraw from the project at any stage.

If you do decide to take part, you will be given this Participant Information and Consent Form to sign and you will be given a copy to keep.

Your decision whether to take part or not to take part, or to take part and then withdraw, will not affect your relationship with your employer nor with the research team or the Stroke Foundation.

6 What are the alternatives to participation?

There are no known alternatives to this implementation project or the education that is provided.

7 What are the possible benefits of taking part?

We cannot guarantee or promise that you will receive any benefits from this research; however, possible benefits may include receiving additional education and support on evidence-based upper limb rehabilitation.

This research will provide a basis for future decisions on the development of implementation strategies for rehabilitation therapists and the support required for their use, which may also in turn greatly benefit the outcomes of stroke survivors (aligned with desired survivor outcomes) in the future.

8 What are the possible risks and disadvantages of taking part?

Participation in this study should involve no physical or mental discomfort, and no risks beyond those of your everyday work life. If you have any concerns regarding anticipated or actual risks or discomforts, please raise them with the Site Principal Investigator.

9 Can I have receive other professional development during this research project?

Whilst you are participating in this research project, you may continue to receive any other professional development; we request that you document the education and professional development you receive, including details such as content, trainer name, total hours of support. The surveys will ask you to share this information.

10 What if I withdraw from this research project?

Participation in this study is completely voluntary and you are free to withdraw from this study at any time without prejudice or penalty. If you wish to withdraw, please contact one of the researchers listed on this form. If you withdraw from the study, the data collected from you will not be able to be removed as it is incorporated without identity to a central database and there will be no way to identify data specific to you.

11 What happens when the research project ends?

After the research finishes, we will employ implementation science methods to work with consumers and clinicians to develop an implementation strategy. Together, these will allow the rapid translation of findings into routine stroke care to improve patient outcomes.
Outcomes from the project will be summarised and shared on the public website of the Stroke Foundation. You may also email Natasha Lannin (natasha.lannin@monash.edu) if you would like to receive a copy of this summary. We anticipate that this summary will be available at the end of 2026 when the research project is completed.

Supplementary File B

Copy of Participant Information Sheet.

Promoting the use of evidence (PROMOTE) in upper limb stroke rehabilitation: multicentre, cluster-randomised, implementation Phase IV trial

Part 2 How is the research project being conducted?

12 What will happen to information about me?

Data from clinicians will be stored in non-identifiable form (coded) and then analysed and reported in such a way that responses will not be able to be linked to any individual or centre. Only members of the research team will have access to the data. All data collected in this study will be stored confidentially on password protected databases and computers. All paper-based documents and signed consent forms will be stored in locked filing cabinets within the locked research office of Natasha Lannin.

It is anticipated that the results of this research project will be published and/or presented in a variety of forums. In any publication and/or presentation, information will be provided in such a way that you cannot be identified, except with your permission.

13 Complaints

If you have any complaints about any aspect of the project, the way it is being conducted or any questions about being a research participant in general, you may contact: Complaints Officer, Office of Ethics and Research Governance, [address; telephone; email]. Please quote the application reference number [local number].

If you suffer any injuries or complications as a result of this research project, you should contact the study team as soon as possible and you will be assisted with arranging appropriate medical treatment. As you are eligible for Medicare, any medical treatment required to treat the injury or complication, will be provided free of charge as a public patient in any Australian public hospital.

14 Who is organising and funding the research?

This research is being conducted by Prof Natasha Lannin and sponsored in Australia by Monash University (sponsor) and is being funded by the Medical Research Future Fund (MRFF).

By taking part in this research project, you agree that data generated from your upper limb rehabilitation may be provided to Monash University. You will not benefit financially from your involvement in this research project even if, for example, your data prove to be of commercial value to Monash University. No member of the research team will receive a personal financial benefit from your involvement in this research project (other than their ordinary wages).

15 Who has reviewed the research project?

All research in Australia involving humans is reviewed by an independent group of people called a Human Research Ethics Committee (HREC). The ethical aspects of this research project have been approved by the HREC of Alfred Health.

This project will be carried out according to the National Statement on Ethical Conduct in Human Research (2007). This statement has been developed to protect the interests of people who agree to participate in human research studies.

The person you may need to contact will depend on the nature of your query.

If you want any further information concerning this project, you can contact the Coordinating Principal Investigator Professor Natasha Lannin via email natasha.lannin@monash.edu.

Supplementary File B

Copy of Participant Information Sheet.

Promoting the use of evidence (PROMOTE) in upper limb stroke rehabilitation: multicentre, cluster-randomised, implementation Phase IV trial

You may also contact any of the following people:

Clinical contact person

Name	Professor Natasha Lannin
Position	Occupational Therapist, Professor
Email	Natasha.Lannin@monash.edu

For matters relating to research at the site at which you are participating, the details of the local site complaints person are:

Complaints contact person

Name	*[Name]*
Position	*[Position]*
Telephone	*[Phone number]*
Email	*[Email address]*

If you have any complaints about any aspect of the project, the way it is being conducted or any questions about being a research participant in general, then you may contact:

Reviewing HREC approving this research and HREC Executive Officer details

Reviewing HREC name	*[Name of HREC]*
HREC Executive Officer	*[Name]*
Telephone	*[HREC Executive Officer Phone number]*
Email	*[HREC Executive Officer Email address]*

Local HREC Office contact

Name	*[Name]*
Position	*[Position]*
Telephone	*[Phone number]*
Email	*[Email address]*

Consent Form - *Adult providing own consent*

**Title**	PROMOTE: A Cluster-Randomised Implementation Trial to Promote Evidence Use
**Project Sponsor**	Monash University
**Coordinating Principal Investigator**	Prof Natasha Lannin
**Site Principal Investigator**	*[Site PI Name and Affiliation Here]*
**Site Location**	*[Location where the research will be conducted]*

Consent Agreement

I have read the Participant Information Sheet or someone has read it to me in a language that I understand.

I understand the purposes, procedures and risks of the research described in the project.

I have had an opportunity to ask questions and I am satisfied with the answers I have received.

I freely agree to participate in this research project as described and understand that I am free to withdraw at any time during the study without affecting my future health care.

I understand that I will be given a signed copy of this document to keep.

Declaration by Participant – for participants who have read the information

Name of Participant (please print) ______________________________________

Signature ______________________________ Date ______________________________

Declaration - for participants unable to read the information and consent form

See Note for Guidance on Good Clinical Practice CPMP/ICH/135/95 Section 4.8.9. A legally acceptable representative may be a witness*.

Witness to the informed consent process

Name (please print) ______________________________________

Signature ______________________________ Date ______________________________

* Witness is not to be the Investigator, a member of the study team or their delegate. Witness must be 18 years or older.

Declaration by Study Researcher†

I have given a verbal explanation of the research project, its procedures and risks and I believe that the participant has understood that explanation.

Name of Study Researcher† (please print) ______________________________________

Signature ______________________________ Date ______________________________

† A senior member of the research team must provide the explanation of, and information concerning, the research project.

Note: All parties signing the consent section must date their own signature.
