# Supplementary material for: Promoting the use of evidence (PROMOTE) in upper limb stroke rehabilitation: protocol for a multicentre, cluster-randomised, phase IV implementation trial
Source: BMJ Open. 2026 Aug 19;16(8):e119551. doi: 10.1136/bmjopen-2026-119551 (PMC13504908; doi:10.1136/bmjopen-2026-119551)
Supplement: Supplementary file 5 [file bmjopen-2026-119551.reviewer_comments.pdf]

PEER REVIEW HISTORY

BMJ Open publishes all reviews undertaken for accepted manuscripts. Reviewers are asked to complete a checklist review form and are provided with free text boxes to elaborate on their assessment. These free text comments are reproduced below.

ARTICLE DETAILS

Title (Provisional)

Promoting the use of evidence (PROMOTE) in upper limb stroke rehabilitation: protocol for a multicentre, cluster-randomised, implementation, Phase IV trial.

Authors

Lannin, Natasha A; Jolliffe, Laura; Scrivener, Katharine; Ada, Louise; Dean, Catherine; Hoffmann, Tammy; Cadilhac, Dominique; Lynch, Elizabeth; Churilov, Leonid

---

VERSION 1 - REVIEW

**Reviewer**	**1**
**Name**	**Chiaramonte, Rita**
**Affiliation and Rehabilitation**	**University of Catania, Department of Physical Medicine**
**Date**	**31-May-2026**
**COI**	**None**

Dear Authors,

The topic is highly relevant, as implementation of evidence-based upper limb rehabilitation after stroke remains a major challenge despite the availability of clinical guidelines.

Here my comments:

Introduction

• I recommend expanding the introduction by discussing the importance of population stratification according to clinical, rehabilitation, socio-economic, and healthcare needs when designing large-scale implementation studies.

• Explain the importance of a multidimensional characterization of patients and healthcare pathways for planning rehabilitation services (doi: 10.3390/healthcare13243200).

• Please better explain what specific knowledge gap this study addresses beyond previous implementation studies in stroke rehabilitation.

• Clarify the expected added value of the PROMOTE package compared with standard audit-and-feedback interventions already described in the literature.

Methods

• Please provide additional details regarding:

o Selection and training of the local champions.

o Standardization procedures across participating centers.

o Monitoring of fidelity to the intervention.

o Potential contamination between intervention and control centers.

• It would be useful to include a figure summarizing:

o Recruitment process.

o Cluster randomization.

o Intervention components.

o Outcome assessment timeline.

• Clarify whether rehabilitation intensity and staffing levels will be collected, as these factors may influence adherence to guidelines and patient outcomes.

Outcomes

• Please clarify how "guideline-recommended upper limb rehabilitation interventions" will be operationally defined and measured.

• Consider reporting how missing documentation will be handled statistically.

• Since the study aims to improve clinical practice, it may be useful to specify whether patient-centered outcomes (participation, quality of life, satisfaction) will be explored.

• Although results are not yet available, I suggest pre-specifying how potential confounding factors will be considered. In particular, it would be useful to discuss whether clinical complications occurring during rehabilitation may be documented, as they could significantly influence functional outcomes and adherence to rehabilitation programs. For example discussing the potential impact of complications during rehabilitation, particularly healthcare-associated infections, the effects of infection of clostridium difficile were describing during stroke rehabilitation (doi: 10.1016/j.idnow.2023.104685). Such complications may prolong hospitalization, reduce therapy intensity, and negatively affect functional recovery, thereby potentially influencing implementation outcomes.

• Please also discuss how findings could be generalized to different healthcare systems and rehabilitation settings.

Strengths and Limitations

• Consider adding a dedicated section discussing:

o Cluster-randomization strengths.

o Potential center-level heterogeneity.

o Risk of contamination.

o Documentation bias.

o Sustainability of the intervention after study completion.

Conclusion

• The protocol addresses a clinically meaningful problem and has the potential to provide valuable evidence regarding implementation strategies for evidence-based upper limb rehabilitation after stroke.

• The manuscript would be strengthened by a broader discussion of patient stratification, healthcare complexity, and factors that may interfere with rehabilitation outcomes.

VERSION 1 - AUTHOR RESPONSE

<table>
<tr><td colspan="2">Reviewer: Dr. Rita Chiaramonte, University of Catania</td></tr>
<tr><td>Dear Authors,
The topic is highly relevant, as implementation of evidence-based upper limb rehabilitation after stroke remains a major challenge despite the availability of clinical guidelines.</td><td>Thank you. We have now actioned your recommendations in our revised manuscript.</td></tr>
<tr><td colspan="2">Introduction</td></tr>
<tr><td>I recommend expanding the introduction by discussing the importance of population stratification according to clinical, rehabilitation, socio-economic, and healthcare needs when designing large-scale implementation studies.</td><td rowspan="3">Actioned.
While the PROMOTE Trial enrols treating clinicians, we have now cite Reviewer 1’s paper in our background discussion regarding the complexity of the stroke rehabilitation inpatient population, and have added the following sentences:
“Importantly, the extent of this evidence-practice gap is not uniform: patients' access to and receipt of rehabilitation varies according to clinical factors such as stroke severity and upper limb impairment level, as well as socioeconomic and geographic factors including rurality and healthcare system capacity [21]. This multidimensional variation in rehabilitation access and delivery underscores the complexity of implementing guidelines at scale and highlights that effective implementation strategies must be responsive to the characteristics of clinicians, the healthcare recipients, and the healthcare pathway.”</td></tr>
<tr><td>Explain the importance of a multidimensional characterization of patients and healthcare pathways for planning rehabilitation services (doi: 10.3390/healthcare13243200).</td></tr>
<tr><td>Please further explain what specific knowledge gap this study addresses beyond previous implementation studies in stroke rehabilitation.</td></tr>
</table>

Clarify the expected added value of the PROMOTE package compared with standard audit-and-feedback interventions already described in the literature.	Actioned.<br>We have now added an additional paragraph to the background:<br>"Existing implementation strategies in stroke rehabilitation, including audit-and-feedback approaches, have shown modest effects on clinician behaviour [24]. However, audit and feedback alone is likely insufficient to drive sustained practice change when barriers to guideline uptake are multifactorial, encompassing gaps in clinician knowledge and skills, limited access to resources, and cultural or organisational factors [23,25]. What has been absent from the literature is a multicomponent, champion-led implementation package that combines education, skill-building, resourcing, and feedback, tailored to the specific barriers identified at each centre, and tested in an adequately powered trial. Our Cochrane review [24] confirmed this evidence gap, finding insufficient evidence to guide which strategies best support clinicians to deliver evidence-based neurorehabilitation."
**Method**	
Please provide additional details regarding:<br>-Selection and training of the local champions.<br>-Standardization procedures across participating centers.<br>-Monitoring of fidelity to the intervention.<br>-Potential contamination between intervention and control centers.	Actioned.<br>We have provided additional details in the TIDieR checklist (Supplemental File C), and in the methods section we have added:<br>"Champions will be employed and selected by site management, drawing from existing experienced clinicians already working within the site."<br>"The risk of contamination between intervention and control centres is minimised by the cluster-randomised design, whereby randomisation occurs at the centre level; however, in the event that staff move between an intervention and a control centre during the trial period, this will be documented and considered in the analysis." "Fidelity to the intervention components will be monitored through champion activity log-sheets, clinician education attendance and module completion records."
It would be useful to include a figure summarizing:<br>- Recruitment process.	Actioned.<br>While Figure 1 already summarises the cluster randomisation and sequence

- Cluster randomization.<br>- Intervention components.<br>- Outcome assessment timeline.	from recruitment, we have now added intervention components and outcome timeline to Figure 1 in line with Reviewer 1's comment.
Clarify whether rehabilitation intensity and staffing levels will be collected, as these factors may influence adherence to guidelines and patient outcomes.	Yes, rehabilitation intensity and staffing levels are collected. We have now added the word "staffing" to clarify this to the existing sentence which explains the data that will be collected on each centre:<br>"Centres will be described by location, hours of therapy per bed (intensity of rehabilitation), funding (private vs public), staffing and caseload volume, and Australasian Rehabilitation Outcomes Centre (AROC) data (e.g. Functional Independence Measure (FIM™) efficiency)."
Outcomes: Please clarify how "guideline-recommended upper limb rehabilitation interventions" will be operationally defined and measured.	As per our method, these are operationally defined by the Stroke Foundation Audit guidelines and referenced; and measured as per our primary outcome (blinded file audit of medical record).<br>We have now added a copy of the operationalised audit criteria as Supplemental File D.
Consider reporting how missing documentation will be handled statistically.	Actioned.<br>We have now added the following under the **Design** section:<br>"Given the nature of the primary outcome, minimal amount of missing data are anticipated. Subject to the satisfiability of missingness at random assumption, depending on the amount of missing data, either complete case analysis or analysis with multiple imputations with chained equations will be undertaken followed by the sensitivity analysis investigating the plausibility of missingness at random assumption. The details will be reported in the statistical analysis plan finalized prior to the study data lock."
Since the study aims to improve clinical practice, it may be useful to specify whether patient-centered outcomes (participation, quality of life, satisfaction) will be explored.	As outlined in our method, patient centred outcomes including quality of life and upper limb performance are measured during the post-implementation phase in all clusters (see page 9 of manuscript).
Although results are not yet available, I suggest pre-specifying how potential confounding factors will be considered. In particular, it would be useful to discuss	We respectfully decline citing the reviewer's paper on Clostridium Difficile infection in our implementation science

whether clinical complications occurring during rehabilitation may be documented, as they could significantly influence functional outcomes and adherence to rehabilitation programs. For example discussing the potential impact of complications during rehabilitation, particularly healthcare-associated infections, the effects of infection of clostridium difficile were describing during stroke rehabilitation (doi: 10.1016/j.idnow.2023.104685). Such complications may prolong hospitalization, reduce therapy intensity, and negatively affect functional recovery, thereby potentially influencing implementation outcomes.	trial on changing practice in allied health staff.<br>We explicitly state that adverse events will be collected and reviewed (which is inclusive of illnesses that require medical attention):<br>"All adverse events will be logged during the trial and rated by site principal investigators for likelihood of trial relatedness prior to being forwarded to the Data Monitoring clinician for review."<br>And we include our DSM details in the TIDieR Supplementary Table (File C).
**Discussion.** Please also discuss how findings could be generalized to different healthcare systems and rehabilitation settings.	We note that there is no Discussion section in BMJ Open protocol manuscripts. Details of the healthcare system and rehabilitation setting have been included in the method (inclusion criteria).<br>We have now also added details re: generalisability to the Strengths and Limitations section (see below)
Strengths and Limitations: Consider adding a dedicated section discussing:<br>- Cluster-randomization strengths.<br>- Potential center-level heterogeneity.<br>- Risk of contamination.<br>- Documentation bias.<br>- Sustainability of the intervention after study completion.	Actioned.<br>In accordance with journal requirements, there is already a required "Strengths and Limitations" section in the BMJ Open manuscript. Therefore, we have now added the additional detail to this section.<br>It now reads;<br>• "This is a fully powered, cluster-randomised Phase IV implementation trial; randomising at the centre level, with matched pairing and concealed, centrally-administered allocation, protects against selection bias and minimises the risk of contamination between arms, although clinician movement between trial centres will be documented and accounted for in the analysis.<br>• The design includes a cost-effectiveness analysis and embedded process evaluation with real-world applicability; the post implementation phase enables examination of clinical outcomes. We acknowledge that sustainability of practice change after withdrawal of the champion and trial resources beyond the 6mo post-

	implementation phase is not being assessed.<br>• Centres vary in staffing, caseload, funding model and baseline guideline adherence; although matched-pair randomisation and pre-specified covariate adjustment will reduce confounding, residual centre-level heterogeneity may still influence implementation outcomes.<br>• The primary outcome relies on medical record audit and may be subject to documentation bias, although blinded assessors and standardised operational definitions will minimise this risk.<br>• All participating centres are within the Australian healthcare system and eligibility requires ≥20 stroke patients to be admitted per year; results may not generalise to smaller, rural or resource-constrained services or non-English speaking contexts, or to healthcare systems with different funding models or staffing structures."
The protocol addresses a clinically meaningful problem and has the potential to provide valuable evidence regarding implementation strategies for evidence-based upper limb rehabilitation after stroke.	Thank you.
The manuscript would be strengthened by a broader discussion of patient stratification, healthcare complexity, and factors that may interfere with rehabilitation outcomes.	Actioned (see above).

VERSION 2 - REVIEW

Reviewer **1**

Name **Chiaramonte, Rita**

Affiliation **University of Catania, Department of Physical Medicine and Rehabilitation**

Date **23-Jun-2026**

COI

I appreciated authors' efforts in improving the manuscript
